# Supplementary material for: A whole-animal phenotypic drug screen identifies suppressors of atherogenic lipoproteins
Source: eLife. 2026 Aug 27;14:RP105314. doi: 10.7554/eLife.105314 (PMC13521566; doi:10.7554/eLife.105314)
Supplement: Supplementary file 2 [file elife-105314-code1.zip › JHDL_primary_result/Myristic acid/Myristic acid_JHDL_17_024_009_2019_09_20_10_01.pdf]

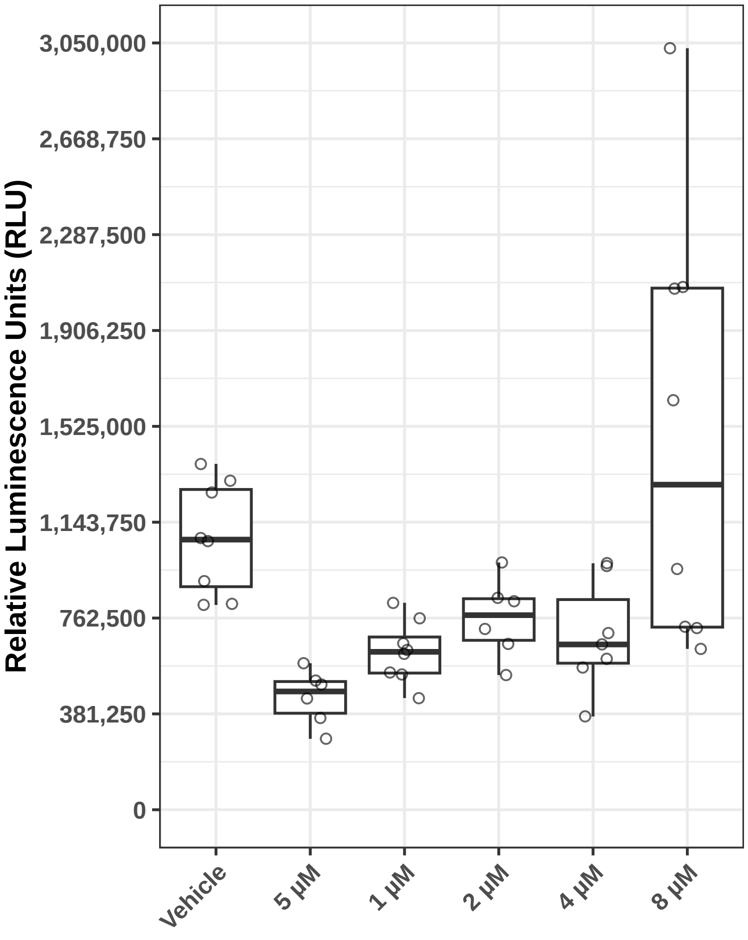
Relative Luminescence Units (RLU)
3,050,000
2,668,750
2,287,500
1,906,250
1,525,000
1,143,750
762,500
381,250
0
Vehicle
5 µM
1 µM
2 µM
4 µM
8 µM
